# Bronchoabsorption; a novel bronchoscopic technique to improve biomarker sampling of the airway

**DOI:** 10.1186/s12931-015-0268-5

**Published:** 2015-09-04

**Authors:** BR Leaker, GC Nicholson, FY Ali, N Daudi, BJ O’Connor, PJ Barnes

**Affiliations:** Respiratory Clinical Trials Ltd., 18-22 Queen Anne Street, London, W1G 8HU UK; National Heart & Lung Institute, Imperial College London, London, UK

## Abstract

**Background:**

Current techniques used to obtain lung samples have significant limitations and do not provide reproducible biomarkers of inflammation. We have developed a novel technique that allows multiple sampling methods from the same area (or multiple areas) of the lung under direct bronchoscopic vision. It allows collection of mucosal lining fluid and bronchial brushing from the same site; biopsy samples may also be taken. The novel technique takes the same time as standard procedures and can be conducted safely.

**Methods:**

Eight healthy smokers aged 40–65 years were included in this study. An absorptive filter paper was applied to the bronchial mucosa under direct vision using standard bronchoscopic techniques. Further samples were obtained from the same site using bronchial brushings. Bronchoalveolar lavage (BAL) was obtained using standard techniques. Chemokine (C-C Motif) Ligand 20 (CCL20), CCL4, CCL5, Chemokine (C-X-C Motif) Ligand 1 (CXCL1), CXCL8, CXCL9, CXCL10, CXCL11, Interleukin 1 beta (IL-1β), IL-6, Vascular endothelial growth factor (VEGF), Matrix metalloproteinase 8 (MMP-8) and MMP-9 were measured in exudate and BAL. mRNA was collected from the bronchial brushings for gene expression analysis.

**Results:**

A greater than 10 fold concentration of all the biomarkers was detected in lung exudate in comparison to BAL. High yield of good quality RNA with RNA integrity numbers (RIN) between 7.6 and 9.3 were extracted from the bronchial brushings. The subset of genes measured were reproducible across the samples and corresponded to the inflammatory markers measured in exudate and BAL.

**Conclusions:**

The bronchoabsorption technique as described offers the ability to sample lung fluid direct from the site of interest without the dilution effects caused by BAL. Using this method we were able to successfully measure the concentrations of biomarkers present in the lungs as well as collect high yield mRNA samples for gene expression analysis from the same site. This technique demonstrates superior sensitivity to standard BAL for the measurement of biomarkers of inflammation. It could replace BAL as the method of choice for these measurements. This method provides a systems biology approach to studying the inflammatory markers of respiratory disease progression.

**Trial registration:**

NHS Health Research Authority (13/LO/0256).

## Background

Robust and reproducible biomarkers may help further the understanding of complex heterogeneous respiratory diseases [1]. Invasive techniques that allow samples to be obtained direct from the airway may have advantages over indirect techniques such as sputum. Current sampling techniques such as bronchoalveolar lavage (BAL) have documented limitations including a lack of sensitivity and specificity [2]. Furthermore, sampling methods should allow comparison of proteomic, transcriptomic and histology data from the same site in the airway. Such data would allow a correlation that would help understand disease severity, different phenotypes and response to drug therapy [3].

Existing techniques for studying respiratory diseases include the analysis of BAL and lung biopsies and non-invasive sample types such as induced sputum and peripheral blood. However even though samples can be collected with relative ease, there are several associated problems [4]. Lack of reproducibility and the effect of dilution on samples collected reduce considerably the accuracy of the inflammatory markers measured. Studies comparing pulmonary biomarkers from different sample types such as sputum, BAL and biopsies from the same individual have shown that there is a large variation in the relative proportion of inflammatory cells in their profiles and a lack of regularity is observed between the different sample groups [5].

There is a lack of direct sampling methods that allow the measurement of markers of disease from specified areas in the airways. In this pilot study we describe a novel sampling technique using absorption of lung lining fluid directly from the site of potential inflammation and compare it with existing BAL techniques in order to compare differences in sensitivity and ease of sampling.

## Methods

The study was approved by the National Research Ethics Service Committee South East Coast (13/LO/0256), and all subjects provided written informed consent. 8 current smokers, aged 40–65 years of age with a ≥ 10 pack year smoking history and a positive smokerlyser test were included in the study.

### Bronchoscopy technique

The bronchoscopy technique was used to collect 3 samples types; bronchoabsorption membrane (synthetic absorptive matrix), BAL and bronchial cytology brushings. Bronchial samples were collected using an Olympus BF-1T20D direct vision scope bronchoscope (Olympus, UK) with an Olympus K-203 guide sheath kit (SG-201C, working length 1050 mm and diameter 2.6 mm, with stopper). Subjects were monitored (vital signs and 3-lead cardiac monitoring) throughout the bronchoscopy procedure and for at least 2 h thereafter. Topical anaesthesia was achieved with the application of lidocaine 2, 4 or 10 % to the nasal passages, (if the nasal route was preferred), pharynx, vocal cords, trachea, the carina, right main bronchus and right middle lobe. The bronchoscope was passed through the nose or mouth with the subject’s head tilted at a 45° angle. The bronchoscope was wedged into a sub segmental bronchus of the right middle lobe. The following order was adhered to throughout the bronchoscopy procedure: bronchoabsorptive matrix, lavage and bronchial brushings.

### Sample collection

Bronchoabsorption exudate - Lung lining fluid was collected using Accuwick Ultra membrane (Pall, USA) which is a specialised fibrous hydroxylated polyester absorptive matrix paper. A pre-cut Accuwick strip was guided through the K-203 guide sheath within the bronchoscope using Olympus forceps (FB-231D, oval, fenestrated swing jaw biopsy forceps, working length 1150 mm, and an outer diameter of 2.0 mm). Strips were left to absorb bronchial secretions for up to 2 min before they were withdrawn back through the bronchoscope.

Bronchial lavage - BAL collection was performed using 4 x 60 mL aliquots (240mL in total) of 0.9 % saline (Nebusal 7 %) pre-warmed. Each aliquot was immediately recovered by gentle negative pressure using a suction pump or directly into a syringe and collected into a container cooled in ice.

Bronchial brushing - Utilizing an endobronchial disposable Olympus cytology brush (BC-202D-2010, 2 mm brush diameter, 10 mm brush length, working length 1150 mm) bronchial brushings were obtained for microarray analysis. A bronchial brush was guided through the K-203 guide sheath within the bronchoscope. Once in position, the brush was gently rotated to collect mucosal cells. The brush was then removed through the guide sheath.

### Sample processing

Bronchoabsorption exudate - Samples were placed in Spin X® centrifuge tube (Sigma-Aldrich, USA) immediately upon receipt and the tube centrifuged at 18620 g at 4 °C for 20 min.

Bronchial lavage - The sample was immediately centrifuged at 480 g at 4 °C for 10 min.

Bronchial brush - A 0.5 mL aliquot of QIAzol lysis reagent (Qiagen, Netherlands) was transferred to tube containing the bronchial brush. The sample was vortexed for 30 s to disperse and lyse the sample.

### Biomarker sample analysis

A total of 13 analytes were measured using Luminex 100 multiplex technology (Luminex Corporation, USA) in bronchoabsorption exudate and BAL samples. The measured parameters were chemokine (C-C Motif) ligand 20 (CCL20), CCL4, CCL5, chemokine (C-X-C Motif) ligand 1 (CXCL1), CXCL8, CXCL9, CXCL10, CXCL11, interleukin 1 beta (IL-1β), IL-6, vascular endothelial growth factor (VEGF), matrix metalloproteinase 8 (MMP-8) and MMP-9 (R&D Systems, UK).

### Gene expression sample analysis

mRNA and miRNA gene expression analyses on the bronchial brushes was performed by Aros Applied Biotechnology (Denmark).

Extracted RNA from the bronchial cytology brushes was analysed using the Agilent Bioanalyser 2100, eukaryote total RNA nano series II (Agilent, USA) to verify the RNA integrity number (RIN). Total RNA was used to synthesise double stranded cDNA which was transcribed to cRNA using *in vitro* transcription and purified, the yield was determined using a Nanodrop (Nanodrop technologies). Second cycle cDNA was synthesised and hydrolysis was carried out using the Ambion®WT expression kit (Ambion, UK). Second cycle cDNA was fragmented and the single-stranded cDNA was labelled using the GeneChip® Whole Transcript (WT) Sense Target Labelling Assay (Affymetrix, USA). The fragmented and labelled single stranded cDNA was hybridized to Affymetrix Human Transcriptome 2.0 Array (Affymetrix, USA) and washed and stained using the GeneChip® Hybridization, Wash and Stain kit (Affymetrix, USA).

## Results

### Bronchoscopy procedure

All subjects were determined to be healthy (smokers with no disease as outlined using GOLD – Global initiative for chronic Obstructive Lung Disease) based on spirometry (Table 1). The bronchoscopy procedure was generally well tolerated with 2 of the 8 subjects reporting minor adverse events post procedure (headaches and chest pain). Sufficient exudate fluid volume was collected from all subjects using the bronchoabsorptive matrix as indicated in Table 2 and was used to perform cytokine analysis. Table 2 also indicates the variability in bronchial fluid retrieval during the BAL process, the volume of fluid collected ranged between 2.75 and 118.5 mL. Bronchial brush samples were not collected from 2 subjects due to early termination of the bronchoscopy procedure. In one instance this occurred due to bronchoscope malfunction. In the other instance the subject presented with anomalous lung anatomy in the right lower lobe, the procedure was completed using a different lobe but due to the added time, the subject experienced coughing and the procedure had to be curtailed.

**Table 1:** Demographic characteristics

	Smoker subjects (*N* = 8)
Gender, n	
Male/Female	6/2
Age (years)	
Mean (SD)	52.6 (11.5)
Range	44–65
Race	
White/Other	6/2
Weight (kg)	
Mean (SD)	82.9 (21.0)
Range	67.5–97.5
Body Mass Index (kg/m^2^)	
Mean (SD)	28.2 (2.7)
Range	21.8–31.1
FEV_1_(L)	
Mean (SD)	3.2 (0.6)
Median (range)	3.3 (2.3–4.0)
FEV_1_ (% predicted)	
Mean (SD)	100.1 (10.2)
Median (range)	100.5 (76.0–112.0)

**Table 2:** Volumes of bronchoalveolar lavage (BAL) and lung lining fluid (exudate) collected by the bronchoscopy procedure

Patient ID	Volume of BAL collected (mL)	Volume of exudate collected (μL)
Subject 1	117.5	42
Subject 2	85	94
Subject 3	105	49
Subject 4	118.5	19
Subject 5	62	75
Subject 6	82	34
Subject 7^a,b^	29	37
Subject 8^a^	2.75	66

^a^Bronchial brush not collected due to excessive coughing, resulting in early termination of bronchoscopy procedure. ^b^Anomalous endobronchial anatomy

### Biomarker analysis

**Fig. 1:**
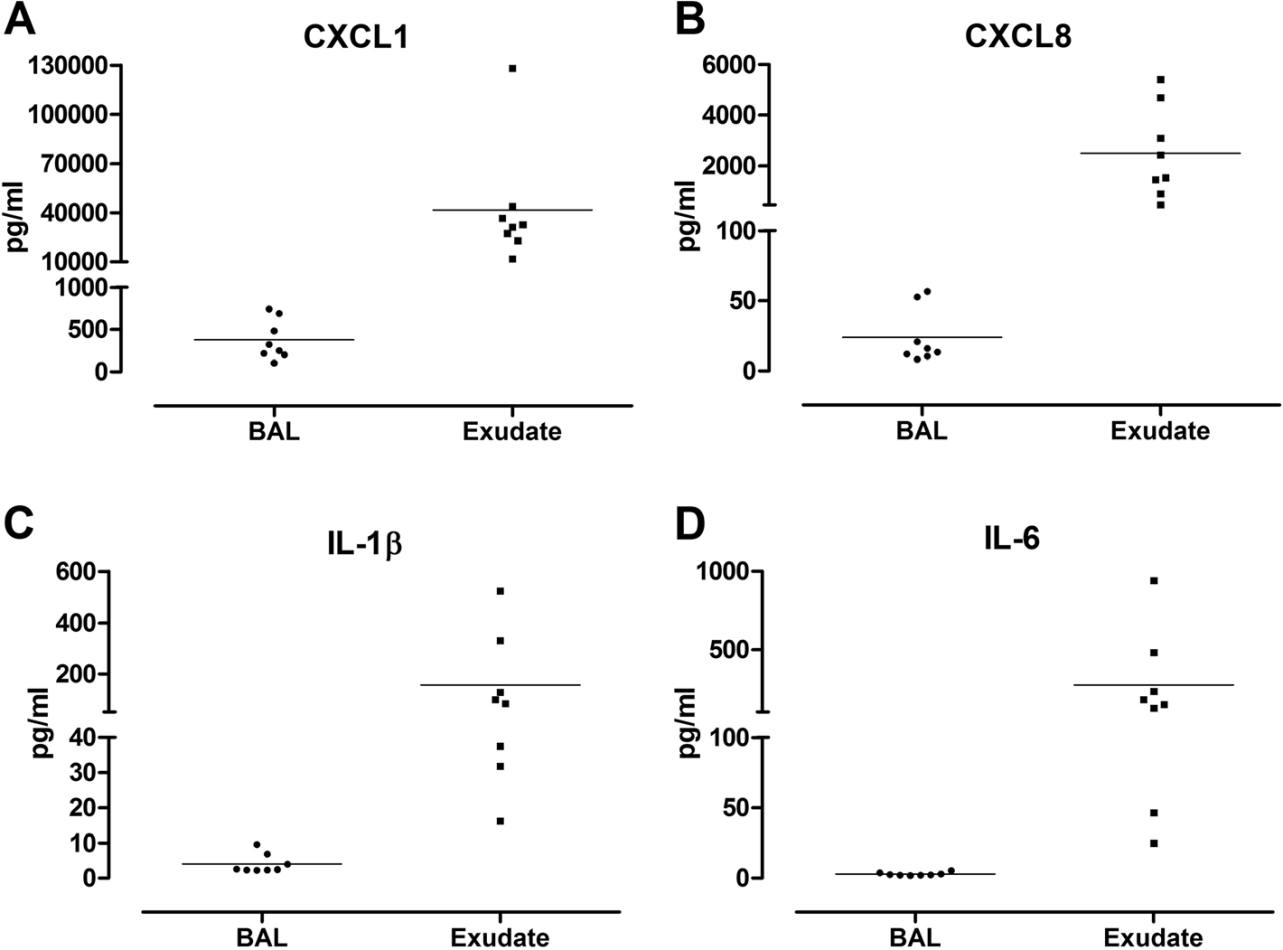
Levels of pro-inflammatory cytokines CXCL1 (**a**), CXCL8 (**b**), IL-1β (**c**) and IL-6 (**d**) measured in bronchoalveolar lavage (BAL) and bronchoabsorptive matrix exudate

**Fig. 2:**
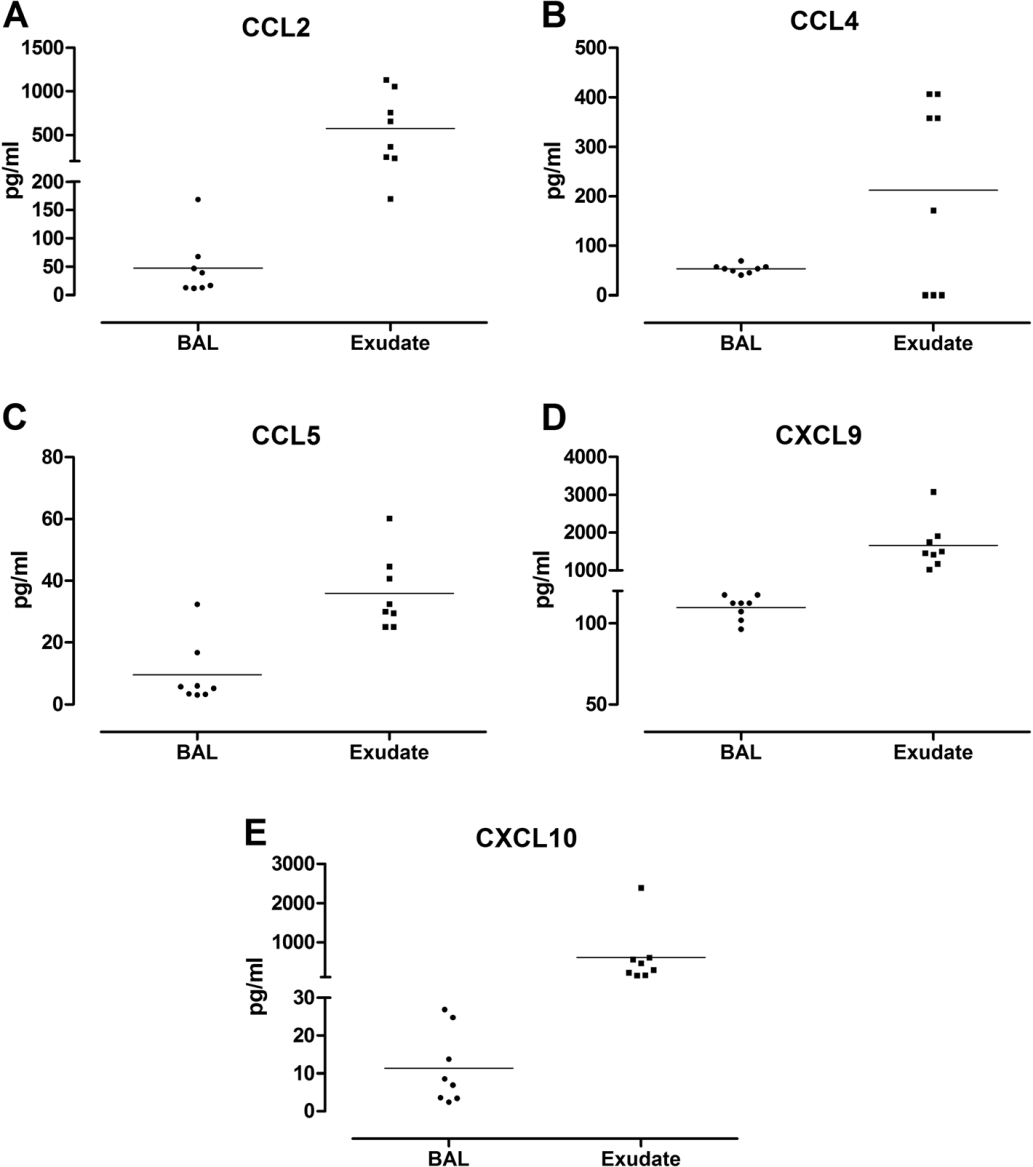
Levels of chemokines CCL2 (**a**), CCL4 (**b**), CCL5 (**c**), CXCL9 (**d**) and CXCL10 (**e**) measured in bronchoalveolar lavage (BAL) and bronchoabsorptive matrix exudate

**Fig. 3:**
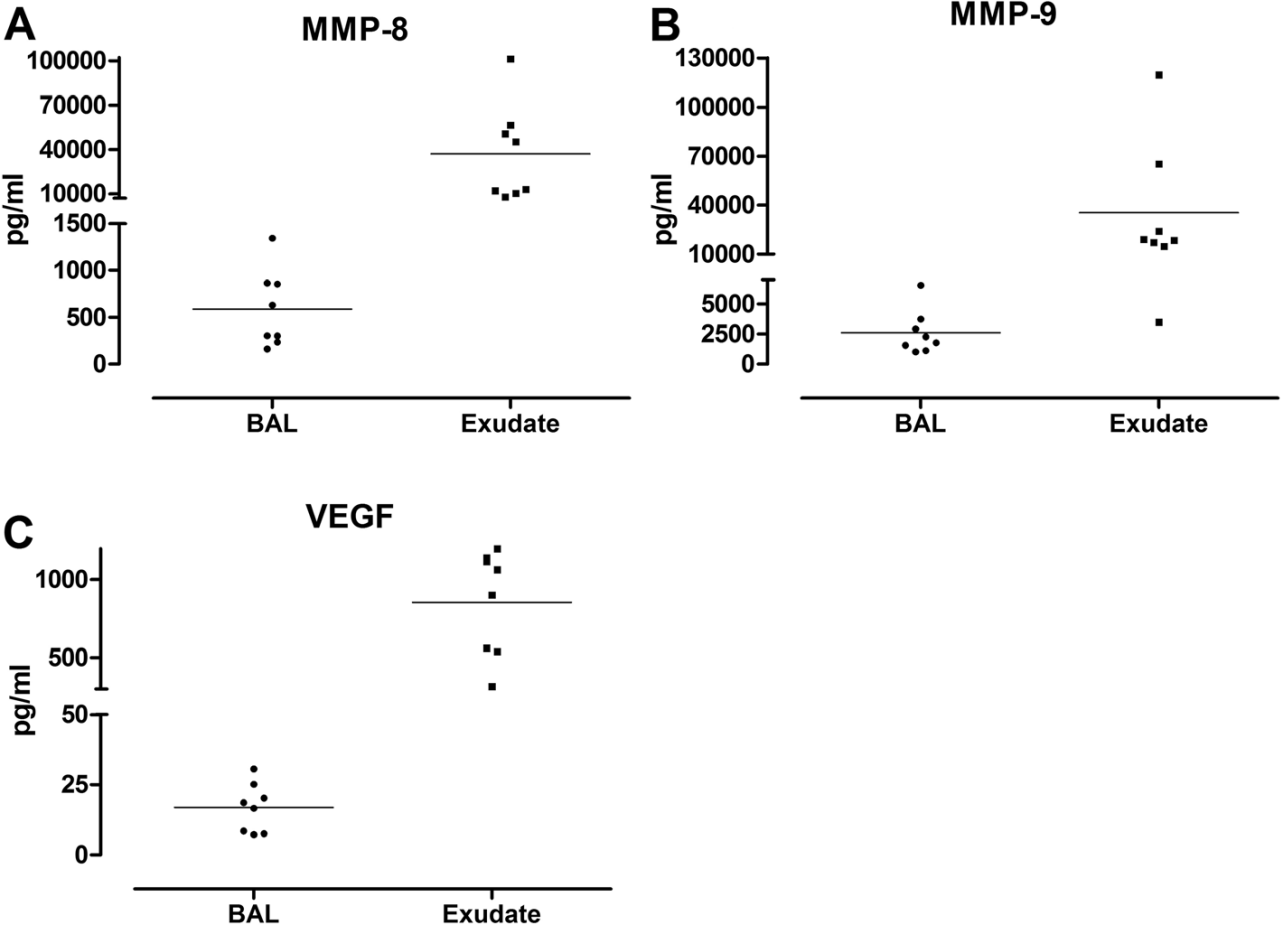
Levels of structural cytokines MMP-8 (**a**), MMP-9 (**b**) and VEGF (**c**) measured in bronchoalveolar lavage (BAL) and bronchoabsorptive matrix exudate

All analytes were detectable, most in both BAL and the bronchoabsorptive exudate samples as shown in Figs 1, 2 and 3. The levels of analytes in BAL measured in this study are comparable to levels of analytes measured in BAL from smokers seen in previously published literature (Table 3). The exudate samples tended to exhibit somewhere in the region of a 10-fold increase in analyte levels compared with BAL samples. Notable exceptions where the concentration in exudate displayed a 100-fold increase compared to BAL were CXCL1 (BAL: 375.96 pg/mL; exudate: 41826.91 pg/mL), CXCL8 (BAL: 23.70 pg/mL; exudate 2496.79 pg/mL) and IL-6 (BAL: 2.89 pg/mL; exudate: 272.74 pg/mL).

**Table 3:** The range of each analyte measured in bronchoalveolar lavage (BAL) and bronchoabsorptive matrix exudate in this study compared to the data range of the analytes found in BAL samples from smokers [35–45], BAL samples from COPD subjects [35, 39, 40, 42, 46–51] and serum samples from smokers [39, 52–56] and serum samples from COPD subjects [39, 48, 57–60] found in literature. All data are pg/mL

	Range in current study		Range in literature			
Analyte	BAL	Exudate	BAL (smokers)	BAL (COPD)	Serum (smokers)	Serum (COPD)
CCL2	11.7–1168.7	170.0–1131.2	4–200	0–150	0–500	0–600
CCL4	40.7–69.1	171.7–406.7	2–23	No data	No data	No data
CCL5	3.1–32.3	25.1–60.3	1–120	1–100	0–15	0–40
CXCL1	103–742.7	11741.4–128091.4	0–10000	0–20	No data	0–50
CXCL8	8.3–56.5	460.4–5395.7	2–141.3	121–2500	0–300	1–1780
CXCL9	96.3–117.5	1018.3–3082	0–800	0–1000	0–1000	0–1500
CXCL10	3.5–26.9	151.3–2393.4	0–175	0–180	147–2541	147–2541
IL-1β	2.2–9.6	16.2–523.7	1–20	0–210	9–15	2.1–28.2
IL-6	1.9–5.3	25–943	1–10	0–40	5–11	1.2–33.8
VEGF	7.2–30.6	317.3–1199.7	0–300	0–130	0–76	151–310
MMP-8	163.3–1344.3	7799–101084.7	16–2330	No data	7000–19000	No data
MMP-9	1128.9–6571.2	3500.9–119543.9	0–5000	17.7–8820	0–6500	0–19000

### Bronchial brush gene expression

**Fig. 4:**
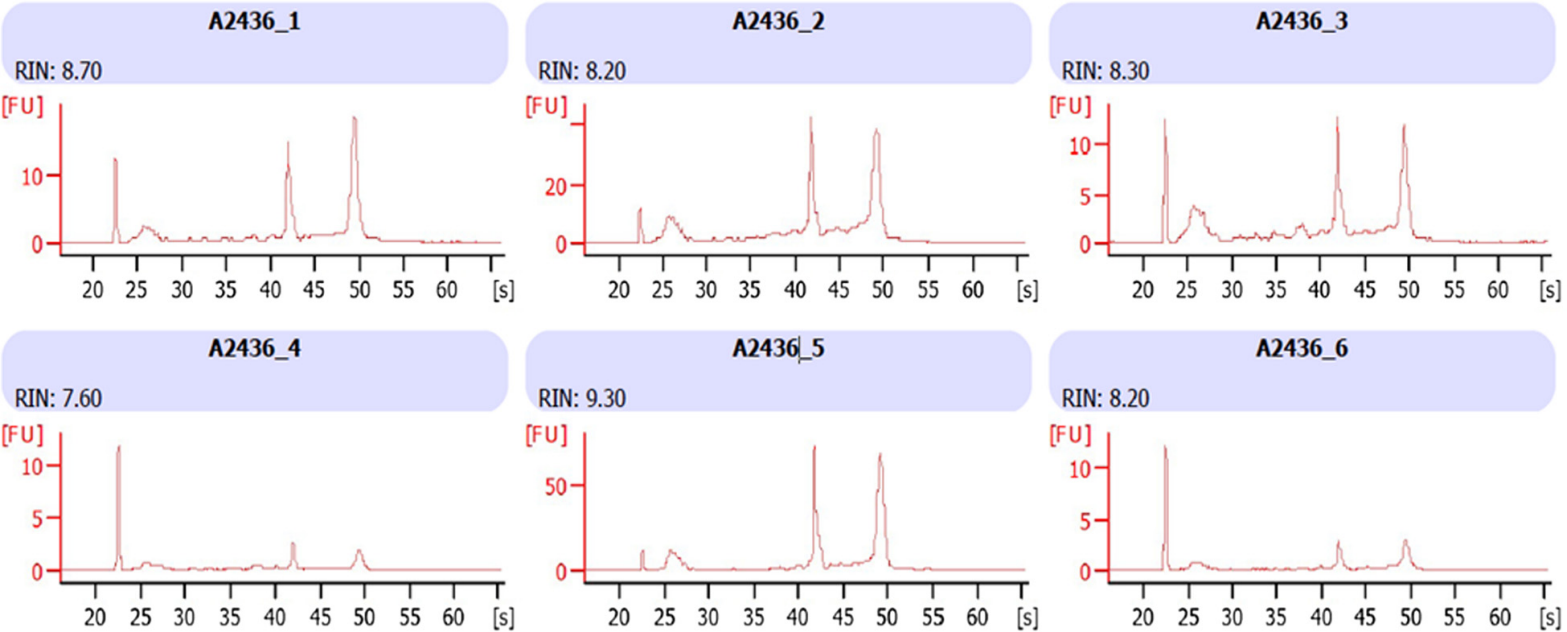
The electropherogram for each of the 6 samples (subject 1- subject 6) analysed using the 2100 Agilent Bioanalyser shows the RNA Integrity Number (RIN) obtained before microarray analysis was performed

Figure 4 shows the RIN for each of the samples analysed. RIN values range from 1 (totally degraded) to 10 (intact). In order to obtain a viable sample with data that can be analysed using cDNA microarrays, the RIN number must be as minimally degraded as possible [6]. The samples analysed following bronchial brush had RIN numbers of 7.6–9.3, indicating that the RNA extracted was of exceptionally high quality.

The gene expression values of the genes CCL2, CCL4, CCL5, CXCL1, CXCL8, CXCL9, CXCL10, IL-1β, IL-6, MMP-8, MMP-9 and VEGF are shown in Table 4, for each of the 6 subjects that bronchial cytology brush scrapes were collected from. The data shows the expression values of a limited number of selected genes. Normally microarray analysis is dependent on the subtraction technique but in this pilot study there was no control group with which to compare the disease state. Nevertheless the data is able to show high yield of good quality mRNA that is reproducible in all the samples. The expression levels for each analyte shown in Table 4 are reproducible across the subjects, with a strong signal that corresponds to the concentration values observed in biomarker data obtained.

**Table 4:** Gene expression values from bronchial brushes. Data are log transformed of fluorescent intensity

Gene	Subject 1	Subject 2	Subject 3	Subject 4	Subject 5	Subject 6
CCL2	8.96	6.84	7.27	6.83	9.14	7.11
CCL4	6.30	6.82	6.32	6.39	6.41	6.48
CCL5	7.43	7.27	8.16	7.60	8.35	7.65
CXCL1	7.84	8.45	8.52	8.62	8.75	8.64
CXCL8	8.47	8.58	8.95	8.89	8.83	8.24
CXCL9	6.17	6.04	6.94	6.91	6.49	6.47
CXCL10	6.91	5.44	7.57	7.74	7.37	8.06
IL-1β	6.74	6.25	6.10	6.22	7.10	6.43
IL-6	5.36	6.03	5.44	5.42	5.35	5.48
MMP-8	3.58	4.76	3.63	3.76	3.47	3.56
MMP-9	6.49	6.98	6.75	6.53	6.59	6.59
VEGF	7.64	7.73	7.10	8.01	7.67	7.98

## Discussion

Chronic obstructive pulmonary disease (COPD) is a common inflammatory disease of the airway and is increasing in incidence throughout the world. The global prevalence of COPD is approximately 10 % among individuals over the age of 40 years. The predominant symptom of COPD is shortness of breath which is persistent and slowly progressive. The clinical symptoms are caused by inflammation in the airways [1, 7, 8].

Tissue obtained from bronchial biopsies or lung biopsies in smokers and patients with COPD has identified key pathological stages in COPD. The inflammatory response is characterized by mucous hyperplasia, infiltration of T cells (Th1 predominant) and increased numbers of neutrophils and macrophages. The progressive fibrosis seen in the small airways is thought to be responsible for the irreversible airway narrowing in patients with COPD [9].

Recent reviews [1, 8] have documented existing biomarkers in COPD patients but criticized the lack of information regarding the variability of measurements and lack of correlation with clinical stage or other physiological measurements. For example in a recent meta-analysis (150,000 patients) only sputum neutrophils, IL-8, serum TNFα, and C-reactive protein were correlated (trends only) with COPD stages [10].

Biomarkers should be easily measurable, reproducible and preferably be straightforward to sample. Recent advances in transcriptomic and proteomic analyses have added to the spectrum of biomarkers available but further studies are needed with repeat measurements in patients and controls where the phenotype has been carefully defined.

The respiratory epithelium is thought to be identical throughout the respiratory tract and similar responses to inflammation have been reported in the upper and lower airways [11]. There are several anatomic similarities between the upper and lower respiratory tracts such as the columnar epithelial cell lining, the mucosal glands and vascularity and innervation as well as the early allergic response seen during asthma and allergic rhinitis suggesting a bidirectional link between the two airways [12, 13]. However it remains the case that bronchial samples obtained directly from the lung are thought to be most representative of inflammation in the lung and biomarkers obtained from nasal samples often thought of as surrogate markers at best [14]. The differences between the nose and lower airways may influence inflammatory responses; for example there is a different vasculature in the nose and in the lower airway. The nose has an extensive system of capillaries, arteries and venous sinuses, an important characteristic of nasal mucosa as changes in vascularisation can cause nasal blockage. However in the bronchi there is a smooth muscle barrier between the lower airway mucosa and vascular supply which does not exist in the nose and is responsible for bronchoconstriction in asthma [15]. Data shows that there are significant differences in gene expression between the upper and lower airways in healthy individuals and that these differences are reduced during allergic inflammation suggesting the inflammation of upper airways also effects the lower airway [16].

Two of the most common techniques used gather samples from the lung are BAL and mucosal bronchial biopsy, and studies using these techniques provide important mechanistic data for the evaluation of anti-inflammatory therapies for asthma and COPD. A European Society Task Force has issued guidelines for measurements of acellular components and standardization of BAL [17, 18] and a similar ATS guideline has also been published to standardise the measurement of cellular components of BAL in interstitial lung disease. The reproducibility of endobronchial biopsy has also been established [19].

There are however inherent problems in the interpretation of samples derived from BAL. By nature BAL fluid collection is variable as several different factors can impact the quantity and quality of the BAL fluid retrieved. Studies have shown that the composition of the BAL fluid collected varies depending on the area the fluid is collected from and this can affect the proteomic profile of the supernatant retrieved [20, 21]. Interpreting BAL findings can be difficult as the procedure cannot be standardised and accurate quantification of the cellular and acellular components in BAL fluid cannot be determined [17, 22]. The causes of variability can be the underlying disease itself, the suction pressure used during aspiration, recovery of lavage fluid, contamination of airways and the area of lung being lavaged [23]. The retrieval volume of the samples is highly variable and can be affected by the age, gender, smoking status and method of retrieval used. The dilution effect on individual biomarkers and unpredictable absorption of BAL fluid from the lung after installation is most pronounced in COPD patients and makes comparison between subject groups and even within patient comparisons difficult [24].

Alternative techniques exist to avoid the problems associated with BAL. Soluble mediators from nasal secretions can be analysed using a filter paper method [25–27]. Strips of filter paper are placed, one on the nasal septum and one on the inferior turbinate so as to allow for absorption of nasal secretions into the matrix of the paper. After removal, the strips are placed into a buffer and soluble mediators are eluted for later analysis. We have determined that nasal samples obtained from the filter paper technique are more sensitive and accurate than those obtained from nasal lavage [28]. Nasal sampling techniques can also be used to gather transcriptomic data directly from the respiratory epithelia. Nasal scrapes and nasal brushings are an ideal tool for collecting genetic material, including mRNA and miRNA, directly from the site of inflammation.

In this study we have adapted the above nasal techniques to obtain relevant samples from the lower airway; bronchial mucosal lining fluid for protein analysis and bronchial brushings for transcriptomic analysis from the same site. Although not performed in this study it would be possible to also collect a bronchial biopsy sample from the same site. The novel bronchoabsorption technique established in this study has addressed many of the issues that arise with BAL by directly sampling the bronchial mucosal lining fluid and avoiding any complications of contamination and dilution.

This pilot study demonstrates the utility of the techniques to harvest lower airway samples. The study also allows comparison of the existing standard technique (BAL) with the novel bronchoabsorption method. Table 2 illustrates the variability of the volume of fluid retrieved after BAL, which becomes even more variable in COPD patients. In contrast exudate levels not only demonstrate less variability in retrieval volume but more importantly the exudate fluid is undiluted. The analysis of each analyte shows at least 10 fold higher concentration in exudate than BAL. This increase in measurable levels increases the sensitivity of the assay markedly and for analytes present at low levels may be the difference in being able to detect the biomarker of interest.

The BAL technique is thought to sample mainly from the distal airway in comparison to the precise anatomical location of the sample obtained using the filter paper technique. It is certainly possible that differences seen in the concentrations of inflammatory markers may be related to where the sample was taken from. However, the respiratory epithelium is uniform throughout the respiratory tract (unified hypothesis [11]) and therefore a similar pattern of inflammatory response should be seen in proximal and distal airways.

The levels of inflammatory mediators are partially genetically determined and information obtained from the genome scale can give information on respiratory disease pathogenesis. GWAS of biomarkers help locate novel genetic determinants and pathways of complex airway diseases and determine the extent of the genetic role on systemic inflammation [29].

In order to perform GWAS, blood samples are collected from COPD subjects and mRNA is extracted to perform gene expression analysis [30, 31]. However systemic markers for inflammation found in blood samples from COPD patients have not been determined as accurate indicators of inflammation in the lungs. Systemic inflammation could be an effect of the inflammation COPD mediators cause in the airways or a separate component of the disease which is independent of the processes occurring in the lungs [32, 33]. Therefore gene analysis of samples representative of the processes occurring in the lungs is required.

This technique allows the collection of direct mucosal samples from the same site as the lung exudate samples which enables gene expression analysis to be performed using the mRNA extracted from bronchial mucosal cells. This systems biology approach of sample collection gives a complete picture of molecular and transcriptomic markers of inflammation that allows for the subtyping of airway diseases. The results reflect the ability of the method used to be able to collect samples of high yield and quality as seen in Fig. 4. RNA integrity numbers of greater than 7 are indicative of good quality genomic samples which would allow the comparison of the transcriptional profiles obtained. Samples obtained using our novel technique ranged from 7.6 up to 9.3. While most microarray data analysis is performed via a comparison with control groups or baseline levels, our study did not include a control group. The data from this study serves to illustrate the viability of the samples collected using this technique and the reproducibility of the expression levels observed of the subset of genes of interest across the patient samples.

Biomarker profiling improves the ability to screen for novel markers of disease to aid with drug development. Treatments focusing on anti-inflammatory agents that target epithelial lung injury and subsequent inflammation are gaining importance and momentum. The approach to future developments of novel drug targets are to stop disease progression and prevent further decline in lung function by blocking inflammatory processes and structural remodelling events taking place in the airways [34]. Therefore targeting the blocking of specific cytokines and chemokines implicated in the inflammatory process may be important in this process.

## Conclusions

The current study has validated a novel direct bronchial sampling technique allowing for the measurement of biomarkers direct from the site of pulmonary inflammation. Using direct vision bronchoscopes the novel method uses a combined approach to collect multiple samples such as biopsies, lung exudates and BAL from the same site to help shed light on a less superficial understanding of different disease mechanisms and builds towards a systems biology approach to understanding the disease. At the same time this technique enables the collection of samples from multiple sites in the same individual using a validated bronchoscopy method without increasing the time or the risk to the patients involved. This procedure can be utilised to compare the responses between varying disease and non-disease states to help understand the pathogenesis of obstructive airway diseases.

## Abbreviations

BAL: Bronchoalveolar lavage
CCL: Chemokine (C-C motif) ligand
CXCL: Chemokine (C-X-C motif) ligand
COPD: Chronic obstructive pulmonary disease
IL: Interleukin
MMP: Matrix metalloproteinase
RIN: RNA integrity number
VEGF: Vascular endothelial growth factor
